# Bovine tuberculosis in youngstock cattle: A narrative review

**DOI:** 10.3389/fvets.2022.1000124

**Published:** 2022-09-23

**Authors:** Andrew W. Byrne, Damien Barrett, Philip Breslin, June Fanning, Miriam Casey, Jamie M. Madden, Sandrine Lesellier, Eamonn Gormley

**Affiliations:** ^1^One-Health and Welfare Scientific Support Unit, National Disease Control Centre, Department of Agriculture, Food and the Marine, Dublin, Ireland; ^2^ERAD, Department of Agriculture, Food and the Marine, Dublin, Ireland; ^3^Centre for Veterinary Epidemiology and Risk Analysis (CVERA), School of Veterinary Medicine, University College Dublin (UCD), Dublin, Ireland; ^4^Nancy Laboratory for Rabies and Wildlife (LRFSN), ANSES, Technopole Agricole et Vétérinaire, Malzéville, France; ^5^Tuberculosis Diagnostics and Immunology Research Laboratory, School of Veterinary Medicine, University College Dublin (UCD), Dublin, Ireland

**Keywords:** mycobacterial infection, calf infection, *Mycobacterium bovis*, diagnostics, gamma interferon, skin test

## Abstract

Bovine tuberculosis (bTB), caused by *Mycobacterium bovis*, remains a high-priority global pathogen of concern. The role of youngstock animals in the epidemiology of bTB has not been a focus of contemporary research. Here we have aimed to collate and summarize what is known about the susceptibility, diagnosis, transmission (infectiousness), and epidemiology to *M. bovis* in youngstock (up to 1-year of age). Youngstock are susceptible to *M. bovis* infection when exposed, with the capacity to develop typical bTB lesions. Calves can be exposed through similar routes as adults, *via* residual infection, contiguous neighborhood spread, wildlife spillback infection, and the buying-in of infected but undetected cattle. Dairy systems may lead to greater exposure risk to calves relative to other production systems, for example, *via* pooled milk. Given their young age, calves tend to have shorter bTB at-risk exposure periods than older cohorts. The detection of bTB varies with age when using a wide range of ante-mortem diagnostics, also with post-mortem examination and confirmation (histological and bacteriological) of infection. When recorded as positive by ante-mortem test, youngstock appear to have the highest probabilities of any age cohort for confirmation of infection post-mortem. They also appear to have the lowest false negative bTB detection risk. In some countries, many calves are moved to other herds for rearing, potentially increasing inter-herd transmission risk. Mathematical models suggest that calves may also experience lower force of infection (the rate that susceptible animals become infected). There are few modeling studies investigating the role of calves in the spread and maintenance of infection across herd networks. One study found that calves, without operating testing and control measures, can help to maintain infection and lengthen the time to outbreak eradication. Policies to reduce testing for youngstock could lead to infected calves remaining undetected and increasing onwards transmission. Further studies are required to assess the risk associated with changes to testing policy for youngstock in terms of the impact for within-herd disease control, and how this may affect the transmission and persistence of infection across a network of linked herds.

## Introduction

Bovine tuberculosis (bTB) remains a priority pathogen of concern in many regions across the globe (1), and especially to cattle industries in Ireland (2) and the UK (3). The national control programmes in developed countries where the disease is endemic relies on the comprehensive ante-mortem testing of cattle and the post-mortem surveillance of slaughtered animals for gross pathology. Detecting bTB infection quickly and imposing control restrictions is paramount in reducing the spread (transmission) of infection within and between herds. Where testing has had to be reduced, for example in Britain due to an outbreak of foot and mouth disease (4), an increased incidence of infection and geographic spread was measured subsequently.

The emerging COVID-19 pandemic in 2020, caused by the virus SARS-CoV-2, had a significant impact on many facets of human society, including animal disease control programmes (5). Agile decision-making processes allowing changes to disease control programmes were required and where possible, informed with rapid analysis of data [e.g., (6)]. One example of COVID-19 impacts in national programmes was on calf testing protocols in Ireland, England and Wales (7). In Ireland, there was a temporary easement for the bTB testing of calves from 42 to 120 days of age in 2020–2021, to allow for suitable social distancing between the veterinary practitioner and farmer during the testing of these calves. This easement was responsible for a facilitation of the movement of calves up to 120 days of age, which benefited trade and was welcomed by the industry. In England and Wales, a temporary easement for calves up to 180 days was facilitated but discontinued in August 2021 (7). Given the epidemiological risks associated with such easements, this narrative review attempts to collate accessible scientific evidence and information on bTB in youngstock, specifically on the susceptibility, exposure, pathobiology, immunology, diagnosis, infectiousness, and epidemiology within this cohort. The paper concludes by identifying gaps in our knowledge and future studies that could help provide an evidence base for future evaluations of the risks associated with youngstock cohorts.

## Are calves susceptible to bTB?

Youngstock are sensitive to *M. bovis* infection (8), and have been used as experimental *M. bovis* infection models in bTB research for several decades [e.g., (9, 10)]. Calves are also the primary host model for bTB vaccine development studies (8, 11, 12). These experimental studies have demonstrated the susceptibility of calves to infection when exposed to a controlled *M. bovis* infectious dose (8). Infection has been established *via* several routes of inoculation (e.g., intra-nasal, intra-tracheal, intra-tonsillar, and aerosol routes) using a wide range of challenge doses [e.g., 10^2^-10^8^; Colony Forming Unit (CFU); see (8)]. Susceptibility to infection has been demonstrated following challenge doses with as low as one CFU of *M. bovis* (13).

Field data derived from infected populations also provide evidence of calf susceptibility to *M. bovis* infection, including the disclosure of calves with bTB lesions (see Pathobiology section) and laboratory confirmed *M. bovis* infection in youngstock. Post-mortem surveillance data of tuberculin reactors in Ireland (14), the UK (15–18), and elsewhere [e.g., (19)], has shown that reactor calves exhibit relatively high lesion disclosure rates at ages of < 1 year. Case studies like Mekonnen et al. (20) demonstrate that calves can succumb to infection under natural conditions very early in life (< 3 weeks of age), with rapid disease progression and associated pathology.

Experimental field trials have demonstrated how calves exposed to reactors in close proximity can become infected within 2–4 months under field conditions (21, 22), and that the susceptibility, or at least the progression of pathological responses, can be reduced when calves are vaccinated with Bacillus Calmette–Guerin (BCG) vaccine.

Youngstock immune response to exposure to *M. bovis* may be impacted by stress factors, including nutritional and transport related stress (23), though the evidence base for this is rather limited. Nutrition and the general health of hosts, including access to the appropriate levels of micronutrients, may also impact host susceptibility (24).

### Take home message

Youngstock are susceptible to infection when exposed to an infectious dose of *M. bovis*, with calves and yearlings capable of developing pathologies as demonstrated by both experimental and epidemiological field data.

## How are youngstock exposed to the pathogen? Age related patterns of exposure

Youngstock may be exposed to infection in herds *via* routes that also affect other age cohorts at the farm level (25). The primary exposure routes of bTB to herds include: 1. residual infection arising from undiagnosed infection, which is apparent after the failure to clear infection following a previous herd breakdown (26–29), 2. infection due to spillback from wildlife (30–32), 3. the buying-in of infected cattle (33–35), and 4. spill-over from infected contiguous herds (36–38), 5. potentially from exposure to *M. bovis* present within the farm environment (39–42).

Youngstock can be exposed as very young calves and contribute to chains of transmission. One case study from Italy described how a 7-day old calf brought into a herd with a recurrent outbreak infection was found to have tuberculous lesions at slaughter 5-months later, possibly because of exposure from infected goats (29). Another example, Rossi et al. (35) also describe the case of an index calf case in an outbreak in Cumbria England, which could be traced back *via* genomic epidemiological approaches to a direct cattle-introduction from Northern Ireland. The analysis showed that spillover occurred into the local badger (wildlife host) population (cattle-to-badger transition), which subsequently spilled back infection (badger-to-cattle transition) but was only detected in wildlife 6 years after incursion to the area.

Within herds, youngstock can be exposed to infection through exposure to herd-mates or *via* pseudo-vertical transmission (e.g., *via* suckling where the pathogen is shed in colostrum or milk) from dams and close contacts (43). The latter is less likely in developed countries with competent control programmes in place to remove infected dams (44). Pseudo-vertical transmission may be an increased risk in dairy than in suckler herd settings, due to the pooling of milk and colostrum (43, 45). An example is described in Doran et al. (46), where a 7-year-old cow with tuberculous mastitis milked to feed calves resulted in 25 of 28 calves, born over two seasons, being identified as bTB reactors.

Given that *M. bovis* is a transmissible respiratory infection, the housing conditions, ventilation, and management of calves in terms of group size and mixing of groups, may be important for exposure rates and within-herd spread (25, 47). Experimental evidence suggests that rates of low air exchange increases the likelihood of transmission and exposure to herd mates (25, 48). Contact patterns may be important for highlighting the differential exposure of individuals within herds (49, 50). Cow-calf interactions have been considered as particularly important linkages across contact networks within cattle herds, potentially acting as influential nodes to facilitate pathogen transmission (49, 50).

### Take home message

Youngstock animals can be exposed to *M. bovis via* several mechanisms, including direct and indirect exposure. These include exposure to residually infected herd-mates post-breakdown, pseudo-vertical transmission, exposure to infected wildlife, and potential environmental exposure for pasture-based systems. There is limited data on the relative importance of each mechanism for youngstock specifically.

## Immunology of bTB in calves

The immune responses induced by infection with *M. bovis* are complex and are balanced to provide a level of immune protection while limiting inflammation and destruction of host tissues (51). The generation of granulomas is a key characteristic of tuberculosis in order to protect the host by controlling dissemination of the invading mycobacteria. These granulomas are composed of organized structures of immune cells including macrophages, neutrophils and lymphocytes. During the course of infection, the structures undergo a process of maturation based upon cellular composition and levels of fibrosis and necrosis (52–55). The granuloma morphology is dynamic and can grow and shrink over time. It is the nature of granuloma formation, determined by host-pathogen interactions that controls disease containment or dissemination, the latter leading to high risk of excretion of infectious bacilli. Individual granulomas within the lung can have different fates, indicating that the local cellular environment rather than systemic responses regulates the outcome of infection at the tissue-level.

Cattle are natural hosts for *M. bovis*, and the disease in bovines is similar to human TB in many aspects of disease pathogenesis and the development of immune responses (56, 57). Cattle studies to identify granuloma-forming responses have demonstrated the presence of various cytokines, chemokines, and enzymes that are typical of these structures in humans and other animal models. An early study involving experimental infection of calves by the intranasal route with intranasal inoculation of 2 × 10^7^ CFU of virulent *M. bovis* identified gross lesions in the lung and tracheobronchial lymph nodes as early as 14 days and microscopic lesions as early as 7 days after infection (58, 59). Though the inoculation dose used was high and unlikely to be encountered under conditions of natural transmission, it did highlight the rate and extent of pathological changes occurring soon after infection [see Mekonnen et al. (20) for a similar case under natural conditions].

The specific factors that lead to containment or progression of bTB are not clearly understood. Central to the key events controlling the cell-mediated anti-mycobacterial response of cattle are T cells, specifically, CD4, CD8, and gamma delta (γδ) T cells. The γδ T cells are a subset of T cells that are functional in both innate and adaptive immune responses and are considered to be a bridge linking these two arms of the immune response. They appear to be critical during the first line of defense against invading mycobacteria and, among other functions, they serve to regulate the downstream acquired immune response (60). The proportion of γδ T cells circulating in mice and humans is relatively low, constituting ~1–5% of the circulating peripheral lymphocyte population (61). In contrast, the frequency of circulating γδ T cells is significantly higher in cattle (and other ruminant species) where they represent 30–60% of the peripheral blood lymphocytes in young ruminants (62–65) and decrease to adult levels after 6 months (65).

The results from other studies in calves have shown that most lymphocyte subpopulations and other functional immune cell functions reached stable levels during the first 6 months of life (66). The implications of these dynamic cell population changes for infection of youngstock with *M. bovis* are not well-understood. New-born calves start their lives with a competent, well-developed but still immunologically naïve immune system, where specific responses against pathogens develop over time (67). At a very young age protective immunity to many pathogens is reliant on passive immunity and transfer of maternal antibodies in colostrum during the neo-natal period. It is unlikely that this will exert a protective response against infectious challenge with *M. bovis* and therefore young calves may be relatively more susceptible to progressive tuberculosis if exposed at a very young age. This idea is indirectly corroborated with surveillance data the shows that skin test positive youngstock have the highest lesion confirmation risk of all age cohorts [e.g., (68)].

Age-related risk factors for tuberculosis in humans have been recognized for many decades. Physicians have known that tuberculosis infections diagnosed in early childhood and in adolescence carry a high risk of advanced tuberculosis. In a key study carried out among children in Puerto Rico between 1949 and 1951, children under 4 years of age had the highest tuberculosis rates. At all ages, children with the strongest sensitivity to tuberculin injection had the highest rates of subsequent tuberculosis (69). The precise reasons for increased severity of TB in young immune-competent individuals is unclear, though as pathogenesis is linked to strong hypersensitivity and the host inflammatory response, this could contribute to the most severe immune responses among younger and healthier age groups (70). In cattle, it has been reported that youngstock can exhibit increasing reactivity responses to tuberculins, as measured during skin tests [e.g., (71)]. It appears cattle reactivity to tuberculins in non-infected cattle increases with age up to 2–3 years and wanes in older cohorts, for example Cagiola et al. (72) reported highest reactions in 2–3-year-old cattle and lowest in 6–7-year-old cattle.

### Take home message

Infection by *M. bovis* in cattle hosts result in a complex immunological response, which is balanced between immune protection and host tissue damage caused by inflammatory responses. Calves have competent, but naïve immune systems, that adapt to pathogen specific responses over time. Given this, it is likely that calves are immunological more susceptible and can progress to more severe disease etiologies than other older cohorts.

## Pathobiology of bTB in calves and lesion detection

As in adults, bTB is chronic in young cattle and similar lesions to adults are observed several weeks after infection (58, 59). The pathogenesis follows several stages post infection, with the first pathological signs of infection requiring intensive careful pathological investigation (often missed during crude inspection surveillance) can occur from ~7 days post-infection, followed by detectable macroscopic gross lesions from day 14 onwards (8, 59). Large scale studies on naturally infected cattle have shown typically low numbers of tissues exhibiting macroscopic lesions in reactors (73).

Animal (infection) model systems where calves were artificially inoculated with virulent strains of *M. bovis* have been used extensively to track the relationship between immune responses and disease progression with the development of typical granuloma (74). Indeed, bTB lesion severity scores for cattle were developed in 5-month-old calves as a model system (55). Experimental studies generally found relationships between the infectious dose exposure, the severity of disease progression, and the levels of shedding from inoculated animals (both young stock and adult cattle), which has been inferred to indicate greater transmission risk (8, 74, 75). As a result of the complex host-pathogen relationship, even during controlled experimentation the levels of disease severity can be highly variable (8). There is a period between exposure to *M. bovis* and the development of well-formed granuloma, sometimes known as a “epidemiological latency” [this is somewhat separate to the latency, reactivating or dormant states exhibited with *M. tuberculosis*, which has been subject to some debate (8)]. During this period, infection may remain undetected because it does not always strongly stimulate peripheral immune responses and results in negative skin tests [“unresponsive period;” (25)]. The bTB epidemiological latency in cattle is estimated to span from 8 days through to 7 years (25, 76) although the latent period may be shorter in calves (25). As a result, infected animals without macroscopic visible lesions may still be infectious (23); indeed, 50–80% of skin test reactors are non-visible lesion at slaughter (23).

Analysis of large field-based datasets, of animals removed from breakdown herds in Northern Ireland (reactors and exposed in-contact animals), on the relationship between age-class and post-mortem metrics of infection have revealed that age-classes around 1–2 years of age had higher probability of disclosure and higher numbers of lesions detected than older cohorts (17, 71). Similar findings suggesting that the youngest reactor cohorts are mostly likely to have visible lesions have been reported in Britain (15, 16, 45). Using surveillance data, Downs et al. (18) showed that the highest post-mortem confirmation probabilities for reactor animals occurred in the 3–6 month and the 6-month−1-year cohorts, respectively. However, in wholly non-reactor populations in Ireland, there was a positive relationship between lesion disclosure and increasing age (77, 78). This latter finding may be explained by the active removal of older reactive cattle in exposed herds through repeated test and slaughter (71). For non-reactive cattle, or potentially cattle which “walled off” infection, survival into older age-cohorts may occur through non-detection. It has been suggested that some of these animals may also be infectious, the so-called “occult” cohort (79).

### Take home message

Infected calves can develop microscopic and macroscopic pathologies soon after exposure (from 7 to 14 days, respectively). The host-pathogen interaction is complex, and even in controlled experimental studies the pathological and disease outcome (e.g., which tissues are infected, severity) can be variable. Experimental studies have shed light on the pathobiology of lesion formation, its resultant disease severity and its nexus with shedding.

## *Antemortem* detection of bTB in calves

The primary antemortem diagnostic tests used to identify exposed cattle is the tuberculin skin test. Where there is a risk of non-specific interference of the test due to exposure to non-tuberculosis mycobacteria, e.g., in the United Kingdom and Ireland, the Single Intradermal Cervical Comparative Tuberculin (SICCT) test is used (23, 80, 81). These tests are often supplemented in national control programmes by the interferon gamma assay in a herd breakdown situation, to improve test sensitivity (23, 80, 81).

The sensitivity and specificity of these tests have been subject to significant scientific research and some debate. The sensitivity of the SICCT test, for example, has been subject to a wide range of estimates, with reviews of field-based estimates varying from 50 to 66%, but with some outlier studies suggesting sensitivities reaching 75–95% for reactors under standard interpretation (23, 82–84). These analyses generally use non-gold-standard latent class approaches, which are less susceptible to bias than older pseudo-gold-standard comparisons, for example the use of visible lesions, histopathology or culture results to indicate infection status of the host. Specificity for the SICCT has been less contentious, with typical values exceeding 99% (95% CrI 0.99, 1.00) [see (84)]. The sensitivity of the interferon gamma test has been reported as typically higher than the SICCT with estimates ranging from 55 to 88%, and lower specificity (97–98%) (82, 83). A recent meta-analysis suggested central values of 67% (95% CrI 0.49, 0.82) sensitivity and 98% (95% CrI 0.96, 0.99) specificity for the Interferon gamma test (84). There is little evidence in the published literature that these tests perform markedly differently in youngstock, at least >42 days old (see below), than in adult animals. Indeed, it is possible that the test will perform in a similar fashion, given that the cellular immunity matures early in young calves (66, 85); strong cellular immune responses have been recorded following vaccination with BCG at birth and at the age of 5 weeks (67, 86).

Following bTB infection, hypersensitivity to tuberculin generally takes 3–6 weeks (21–42 days) to develop a detectable response (23). Calves younger than 42 days will generally remain test negative [but not always; e.g., (20)], in spite of developing infection. Accordingly, for the rules agreed across Member States in the EU, there is a requirement for all animals over 42 days old to be tested [note, the methodologies including test interpretation are described in the former EU trade Directive 64/432/EEC, which is now superseded by Regulation (EU) 2016/429 (Animal Health Law), and also by the OIE; (87)]. The interferon gamma test is generally restricted to animals over 6 months of age, due to the risk of non-specific responses (81, 86).

Data from reactors culled during bTB breakdowns in Northern Ireland suggested that the confirmation of TB infection was superior in young animals (more than 42 days old) compared with in older animals when based on ante-mortem immunological tests and post-mortem examination. Age-classes around 1–2 years tended to give larger mean comparative tuberculin skin test reactions than older age-cohorts (17). Animals aged 6 months−2 years had a lower probability than older age cohorts of being false negative by SICCT, or SICCT/interferon gamma tests interpreted in parallel, relative to confirmed positive *M. bovis* cultures (88). A Spanish study found a general increase in risk of false negative tuberculin test results associated with age, based on either single intradermal test (SIT) test at severe or standard interpretation, and if using the Interferon gamma test on its own or in parallel with the SIT tests (89). In that study animals under 2 years of age had the lowest ante-mortem false negative test risk (89). Gormley et al. (90) found that animals aged over 5 years were 1.4 times the odds of being a false interferon gamma positive, relative to animals aged 6-months to a year of age [c.f. (91)].

In a prospective study from Great Britain, when disclosed as test positive, the detection of bTB in tissues (either through the presence of lesions and/or laboratory confirmation) was highest in calves/yearlings relative to adult cattle (heifers, sucklers, finishers) (18).

Post-mortem surveillance for disease suffers from poor sensitivity for the detection of infection (81, 83) with large variation between individual slaughter plants (77, 78, 92). However, it still provides a useful diagnostic tool for case detection and surveillance in very low prevalence countries (93) and for missed infection more generally (94).

### Take home message

Overall, the evidence suggests that the detection of bTB infection is most sensitive and specific in calves (more than 42 days old) compared with older animals (more than 2 years of age) by tuberculin skin testing and the interferon gamma assay.

## Infectiousness and transmission of *M. bovis* infected calves

Much of what is known about infectiousness, or the transmissibility of infection between hosts, has been gained from controlled animal experiments (25, 58). Such experimental work has suggested that animals shed pathogens (a prerequisite for transmission), based on the detection of *M. bovis* in nasal mucus or feces, as soon as 11 days or as late as 100 days after inoculation (25, 95). Other studies have shown that 7–8-month-old calves (albeit inoculated with high doses) are capable of transmitting infection to in-contact housed cattle within 28 days (58).

There is a transmission risk to other hosts from infected but ante-mortem test negative animals (23, 95, 96). For example, in a study of 24 experimentally infected calves, two of 15 confirmed shedders had no visible lesions at postmortem examination, one of which was also skin test negative (95). Menzies and Neill (96) review transmission studies and other data highlighting this risk in cattle-to-cattle transmission. Anergic effects from repeated short-interval testing on skin test performance (reducing sensitivity), especially in older animals, could also impact this risk (97, 98).

There is a significant body of experimental work on natural transmission of infection and its modulation *via* BCG vaccination in controlled conditions (99–101) and field trials in calves [e.g., (22)]. Details of the development and application of BCG is beyond the scope of this review. However, in brief, these studies have demonstrated that BCG can have significant long-lasting protective effects on calves (21, 102), including reducing pathological progression, with some authors suggesting that BCG could limit transmission by reducing the numbers of bacilli being produced and shed by the host (103).

Infected calves, if left undetected and untraced, can pose a significant risk for the spread of infection (20, 104). As an example, Francisco et al. (104) documented the spread of infection due to calves in the United States. A culled dairy cow was found to be tuberculous at slaughter, and a back-trace epidemiological investigation revealed a large outbreak in the index herd including 15 animals < 2 years old. Five additional herds were found to have infected cattle, all of which had received calves from the index herd (104). The report concludes that “neonatal calves can have an important role in the transmission of *M. bovis*.”

### Take home message

There are good experimental data demonstrating that infected youngstock are capable transmitting infection to conspecifics, often after relatively short periods post-inoculation. Transmissibility is possibly linked to pathological progression and related to shedding patterns from the infected host. Infection from calves-to-calves and to older cohorts have been reported in farm settings.

## Epidemiological risk factor and modeling studies

Epidemiological studies have shed light on the spread and maintenance of infection across herds in age-structured populations. Humblet et al. (44) reviewed age as an animal-level risk factor for bTB diagnosis. They suggested, based on data reported from Tanzania, Chad, Zambia and Ireland, that ante-mortem test positivity rates increase with age; citing a paper by Griffin et al. (105) reporting that older cattle (cows, heifers, bullocks) were at significantly higher risk failing a tuberculin test than calves. More recent work from Ireland, using a survival model, also found a reducing time to bTB disclosure with increasing age (106). It should be borne in mind that it is possible that ascertainment bias could be a factor in certain situations, such as when calves have low testing frequency. It should be less of a factor in countries with well-developed eradication systems where whole herd tests include all animals >42 days.

Broughan et al. (45) undertook to review the evidence for age as a risk factor for bTB diagnosis. They again reported that there was a general consensus for increasing risk of infection (test positivity) with increasing age, with the relationship described as monotonic, linear or u-shaped across studies reviewed.

Despite these risk factor findings, when risk of recurrence was modeled after the disclosure of an inconclusive skin test finding, it was younger cohorts that were at greatest shorter-term future risk of failing a follow-up bTB test (107), meaning early indicators of infection within younger cohorts may be important for early identification of infected animals in herds.

The movement of animals has been shown to be an important contributor to the maintenance of *M. bovis* infection across herds and populations (108, 109). National level data has illustrated the connectivity and volumes of movements of cattle [e.g., (110–112)]. Such data inform on the potential for bTB inter-herd spread risk. However, such network modeling has yet to be used to explicitly model the risk of bTB maintenance and spread attributable to calves and youngstock. In terms of youngstock trade volumes, Tratalos et al. (111), using Irish data, reported that 28% of all calves undertook a move within their first 7 months of life, increasing to 43% by the age of 10 months. Excluding moves to export, knackery and slaughter, 19% of calves moved to another breeding herd within 10 months of age. Prior research has shown that movement history can be an important factor in cattle bTB risk, with animals moving from dairy herds with recent histories (< 7 months) of large outbreaks having significantly increased risk of failing a bTB test (33). There was a trend in that study for detection risk to decrease with age, but this was dependent on the sex of the animal, and the fixed term was non-significant [odds ratio: 0.98 (95% CI: 0.96–1.00) for each increasing month of age; (33)]. This follows a broader trend of increasing risk for animals sold out of herds with a recent bTB breakdown history (104, 113, 114).

Brooks-Pollock et al. (15) undertook a comprehensive modeling study to investigate the relationship between animal-age and bTB risk using data from Britain. This analysis showed how detection, based on age-structured reactor numbers, increased steeply with increasing age to a highest point around 1–2 years old. The pattern of age-related detection probability was different between beef and dairy animals, however for younger animals under 1 years of age the normalized reactor rates were approximately equal. Brooks-Pollock et al. (15) used these data to develop a simple catalytic mathematical model to estimate the force-of-infection (the rate of susceptible cattle becoming infected) across age cohorts and herd types (beef and dairy). The model demonstrated how the age-dependent bTB risk was higher for the 0–1 year old cohort than 3 years and older cohorts for beef animals. Alternatively, the youngest cohort in dairy herds had the lowest age-dependent risk. Across both production types, the highest risk was associated with age-cohorts around 1–2 years. Their model suggested that on average the mean age at detection was 5–8 months after the mean age of infection. The authors speculate that youngstock have lower chance of detection, but higher risk of being high shedders of mycobacteria and therefore could be an epidemiologically important cohort. Another related model, developed by Conlan et al. (16), also proposes that the lowest risk, based on the ratio of the estimated force-of-infection across age cohorts, was for both beef or dairy animals aged < 1 year. This risk profile increased in this model up to 36 months, falling, then plateauing for animals >5 years of age (16).

A risk assessment was undertaken in the Netherlands to estimate the importation risk of bTB, and what testing approaches were most cost-effective to mitigate this hazard (115). This study suggested that the importation of calves from higher risk trading partners posed the greatest incursion risk, with an estimated 99 positive animals being imported annually, 98% of which being calves. However, very few of these calves ever get detected with bTB—in the preceding 15 years prior to the study, only 23 animals were found test positive. While early detection would benefit from a scheme aimed at more additional testing of higher risk groups (e.g., calves), the additional challenge and cost of testing calves and because they are for veal, the authors highlight it may be more economically feasible to test cows (115).

Schiller et al. (81), identified the movement of untested calves as one of four major routes that bTB is spread by trade. The authors point out that using the current rule sets and diagnostics available, there is an age limit (6 weeks/42 days) for internationally traded animals, below which they are exempt of testing, resulting in the introduction of undetected infection. The authors pose the question whether this exemption should be reviewed.

What is missing from the above analyses are quantitative estimates of the contribution calves make to the maintenance of infection across networks or national herds. On sound epidemiological principles, reducing the number of tests being undertaken is likely to increase the probability of missed detections [e.g., (79)], and by extension, the possibility of intra- and inter-herd spread/maintenance. A recent example is the suspension of testing in Britain during the foot and mouth outbreak, resulting in increases in bTB transmission amongst herds (4). A lack of frequent or adequate testing can result in unknown infections being maintained within herds, with the potential for spillover/spillback to and from wildlife [for example see Rossi et al. (35)]. Each cohort of births represent a new batch of animals susceptible to infection. The role that calves play in infection maintenance and spread has been somewhat understudied, with calves being ignored in some mechanistic modeling studies [e.g., (116)] and statistical investigations [e.g., removal of movements of calves < 42 days old in Clegg et al. (117)]. Indeed, it has been pointed out recently that the presence of infectious calves need to be more carefully considered to ensure that the “appropriate structure of models [are] used to estimate the rate of transmission within herds” (20).

An exception is the model of Risso-Picasso et al. (118) that investigated control strategies for bTB in cattle herds in Uruguay. The compartmental mathematical model split the population into two major groups, calves and adults, with control strategies being only applied to the adult population. Across several model scenarios, the model found that time to outbreak eradication was shorter for adults than for the calves across herds, primarily as calves remained infected in-herd until they reached an age where they could be tested and detected (including lags in hypersensitivity). The model highlighted how calves were responsible for maintaining infection for longer periods within the herds. In the conclusion to their work, the authors highlight the “importance of targeting surveillance and control strategies to infected calves.”

### Take home message

Epidemiological modeling using several analytical approaches has identified increasing age as an important risk factor for animal-level bTB diagnosis. Mathematical modeling has suggested that the force of infection varies across age-cohorts, but this pattern may depend on the herd production type. Network modeling has highlighted how youngstock can be an important factor epidemiologically linking herds *via* trade. Younger cohorts may have lower exposure and disclosure rates, but could contribute to transmission through non-detection, and prolong outbreaks where inadequate testing exits.

## Knowledge gaps and uncertainties

One gap in our knowledge, is the detailed understanding of how tests performance may vary by age-cohort, especially youngstock. It is known that very young animals may not mount an immunological reaction (hypersensitvitiy) to tuberculins early in life, and hence the preclusion of testing for animals under 42 days. Reactivity then increases with age, affecting specificity in disease free situations Reciprocally, the likelihood anergy may increase with age. However, there is some uncertainty what the true sensitivity and specificity of the screening tests are for youngstock, and whether they differ to adult cattle.

Sound epidemiological principles suggests we may expect differential exposure to, and from, calves with other herd mates, potentially governed by contact-patterns (49, 50). However, we have little information on the role of such heterogeneous contact patterns on *M. bovis* spread, or whether calves may act as an important linking cohort between nodes within herd contact patterns.

A significant uncertainty with the current assessment of risk attributable to calves and their testing results data, is the fact that there are delays in the time from exposure, to infection, through to detection due to both biological reasons (latency) and the structure of disease control management programmes (timing of tests). For example, the estimate from Brooks-Pollock et al. (15) is for a 5–8-month delay in detection after infection. Therefore, it is difficult to attribute an infected/infectious status using empirical data to calves. Instead, from an epidemiological perspective, fitting mathematical, and simulation models using retrospective analysis of real-world data may be better tools to truly estimate the significance of calf number, infectious status, and testing regime.

Such models can provide insight into the timing of disease incursions into herds, and the relative contacts or risk calves can play in certain systems (15, 93). Mechanistic models can also provide evidence as to how control strategies can affect the time to eradication of outbreaks, or how lower risk cohorts like calves can maintain infection when untested (118). Currently, there are very few dynamic models that have been utilized to ask “what if” questions around the changing of testing policies for calf or youngstock testing. Furthermore, there are limited risk-assessments to quantify what the likely outcome may be to changes in testing policy. Reasonably simple approaches modeling the risk of calves within herds being exposed, infected, untested, transmitting and moving across a trading network, parameterised with estimates from real world data could be insightful. However, estimating some parameters (like occult infectious status) might be challenging.

Tests that are less reliant on immunological response, e.g., direct detection, would be an extremely useful advance and tool in our armory, but thus far there are limited number of such tests available (81) or uncertainty regarding their test performance. Direct detection may allow for closer to “real time” diagnosis, and may facilitate earlier testing of certain at-risk cohorts, such as youngstock.

## Conclusions

Youngstock represent a replenishing cohort of susceptible animals that are at-risk of infection, especially in herd situations where residual infection exists post-restriction, where wildlife may introduce infection into a herd, where contiguous spread occurs from neighboring herds, or through the buying-in of undetected infected animals. The current standard test diagnostics preclude the accurate testing of very young animals (stipulated to be < 42 days). Youngstock are less likely to test positive than older animals, but there may be a delay in some instances between exposure and detection; exposed animals may only be detected after leaving this age-cohort. Despite this, every year youngstock animals are disclosed as reactors during national control programmes in endemic regions. Youngstock, when test positive, have the highest rates of post-mortem confirmation. This could indicate that they are mounting a strong reaction to pathogen exposure, though lesion development has been correlated with increasing shedding of pathogen. It is probable that the true number of infected youngstock may be higher than the number of test positive animals, as both the screening and ancillary tests for bTB have moderate to poor sensitivities (see above). A high proportion of animals move during the first few months of life; many going on to breeding herds. There are sound epidemiological principles, and recent experiences, that would suggest reducing testing across age-cohorts or frequency would increase the risk of non-detection of infection in populations where the disease is endemic. There is a dearth of modeling studies to inform on the likely magnitude of effects of lowering the amount of testing for particular cohorts on disease levels and control programmes. One paper points to a risk of increasing epidemic duration if calves are not included in bTB control programmes. It is recommended that further modeling work is undertaken, especially to allow for “what if” scenarios to be tested, in the presence of uncertainty and unobservable dynamics (e.g., occult infection presence).

## Author contributions

AWB, DB, and PB: conceptualization. EG, SL, AWB, MC, and JMM: investigation. DB and JF: supervision. AWB and DB: project administration. AWB and EG: writing—original draft. AWB, DB, PB, MC, JMM, JF, SL, and EG: writing—review and editing. All authors contributed to the article and approved the submitted version.

## Funding

Open access publication fees were received from the Department of Agriculture, Food and the Marine (DAFM), Ireland.

## Conflict of interest

The authors declare that the research was conducted in the absence of any commercial or financial relationships that could be construed as a potential conflict of interest.

## Publisher's note

All claims expressed in this article are solely those of the authors and do not necessarily represent those of their affiliated organizations, or those of the publisher, the editors and the reviewers. Any product that may be evaluated in this article, or claim that may be made by its manufacturer, is not guaranteed or endorsed by the publisher.
